# DNA damage by radiation as a function of electron energy and interaction at the atomic level with Monte Carlo simulation

**DOI:** 10.1016/j.zemedi.2022.07.003

**Published:** 2022-08-13

**Authors:** Youssef Lamghari, Huizhong Lu, M'hamed Bentourkia

**Affiliations:** Department of Nuclear Medicine and Radiobiology, University of Sherbrooke, Sherbrooke, QC, Canada

**Keywords:** Monte Carlo simulation, GEANT4, Cancer, DNA strand breaks, Low energy electrons, Dissociative electron attachment

## Abstract

In radiotherapy, X-ray or heavy ion beams target tumors to cause damage to their cell DNA. This damage is mainly induced by secondary low energy electrons. In this paper, we report the DNA molecular breaks at the atomic level as a function of electron energy and types of electron interactions using of Monte Carlo simulation. The number of DNA single and double strand breaks are compared to those from experimental results based on electron energies. In recent years, DNA atomistic models were introduced but still the simulations consider energy deposition in volumes of DNA or water equivalent material. We simulated a model of atomistic B-DNA in vacuum, forming 1122 base pairs of 30 nm in length. Each atom has been represented by a sphere whose radius equals the radius of van der Waals. We repeatedly simulated 10 million electrons for each energy from 4 eV to 500 eV and counted each interaction type with its position x, y, z in the volume of DNA. Based on the number and types of interactions at the atomic level, the number of DNA single and double strand breaks were calculated. We found that the dissociative electron attachment has the dominant effect on DNA strand breaks at energies below 10 eV compared to excitation and ionization. In addition, it is straightforward with our simulation to discriminate the strand and base breaks as a function of radiation interaction type and energy. In conclusion, the knowledge of DNA damage at the atomic level helps design direct internal therapeutic agents of cancer treatment.

## 1 Introduction

According to the National Cancer Institute (NCI), the new cases of cancer were estimated at 1,806,590 in the United States in 2020. This number is expected to increase in the future due to population aging and the availability of modern diagnostic technologies. Apart from the mortality and morbidity of individuals with cancer, the expenditure for cancer care reached 150 billion $ in 2018 in the United States as reported by the NCI. Cancer affects the population at all ages and there are several types of cancer, and all show the rapid proliferation of cancer cells. If diagnosed at an early stage where the tumors are locally confined, the surgery and/or the radiation therapy are the mostly prescribed.

Radiation therapy of cancer starts with imaging with computed tomography (CT) to locate the tumor and to determine its volume. Radiotherapy clinicians and medical physicists work together to establish a treatment plan to target the tumor with radiation and avoid irradiation of sensitive normal tissues. Several radiation beams may be proposed at different entries in the patient. For example, a brain tumor may receive 70 Gy in 7 weeks (35 days), with 2 Gy/day from five photon beams of 6 MeV. Before the start of the treatment, a positron emission tomography (PET) image is taken to estimate the tumor metabolism with 18F-Fluorodeoxyglucose (18F-FDG), then six months after the end of the treatment, another scan with PET-18F-FDG is scheduled to check the tumor response to the treatment. The fractionation of the dose was justified to allow the irradiated normal cells to repair their lesions among other factors, while, recently, the approach of tumor irradiation with flash radiotherapy was reported to be a promising technique.

In radiotherapy, either by an internal or external radiation source, the objective is to damage cancer cells in a solid tumor by breaking their DNA molecules. The particle beam energy and fluence are adjusted in order to deposit the required dose at the tumor site. However, the secondary low energy electrons are the mostly involved in DNA damage. For this reason, experimental and Monte Carlo simulation studies focus on low energy electrons.

Until now, cancer treatment by radiation has been carried out without distinction between subjects, e.g. similar tumor types receive similar radiation doses. However, the physical aspects are nowadays mostly addressed, such as the specification of the beam energy and fluence, depending on the size and location of the tumor. In this regard, Monte Carlo simulations considerably contributed in sparing normal tissues from radiation.

Some Monte Carlo algorithms were compared and relative differences in absorbed dose were found between 5% and 10% for photon energies above 1 keV [1], [2]. The difference could be more pronounced at electron energies below 20 eV since several particle interactions occur at these energies. Furthermore, these comparisons were not performed as a function of the electron interaction types.

The damage to DNA is achieved when the molecular bonds in DNA strands or in DNA bases are broken by the radiation. These damages are not yet understood by which types of electron interactions and by which level of energies they are caused. Most simulations with Monte Carlo consider a DNA strand break to occur based on deposited energy provoking excitation and ionization in water models [3], [4], [5]. Others, more sophisticated, used an appropriate density of DNA [6]. In recent years, DNA atomistic models were introduced but still the simulations consider energy deposition in continuous volumes of DNA material [7], [8], [9].

The Monte Carlo simulations use different platforms and algorithms to simulate radiation damage to DNA, however, the physics of radiation interaction with DNA mainly remains the same depending on the low energy threshold and types of interactions included in the simulations [10], [11], [12]. Apart from the direct interaction of the incident radiation beam and the secondary electrons set in motion in the medium, there are also the water products, especially the water radicals, which contribute to DNA damage [13], [14], [15]. Other simulations concentrated on Auger emissions impacting DNA as some isotopes emit high numbers of Auger electrons such as I125 and Tc99m suitable for internal radiotherapy [16], [17]. Even the DNA repair has been considered in the simulations [18], [19].

Despite the great progress in the simulation of the atomistic DNA and radiation damage to DNA, there remains hot topics still under investigation, among them the physics of low energy electrons below 5 eV, the types of interactions provoking DNA strand or base breaks, simultaneous effects of incident radiation and secondary electrons, environment of DNA, etc. While the experiments can provide the number of DNA breaks irrespective of the interaction types, the simulations can reproduce the experimentations, and they can further provide the DNA breaks as a function of radiation interactions. In other words, the simulations allow to decompose the experimental results into their sub-effects including the possible extraction of the cross-sections.

The development of computing codes dealing with radiation transport, and its importance in biology, led researchers toward the development of digital models of DNA. Among these developments, GEANT4-DNA offers the great advantage in the choice of DNA breakage mechanisms according to the types of interactions and the deposited energy. In this context, several studies have been carried out with Monte Carlo simulations to reproduce experiments reported to measure the dose necessary to induce a DNA single strand break (SSB) and double strand break (DSB) [5], [15], [20], [21]. In addition, some algorithms have been designed to convert the energy deposited into numbers of DNA breaks such as in GEANT4-DNA (PDB4DNA) [7].

In order to develop a simulation tool for internal dosimetry estimation, and in a second step for both internal dosimetry and imaging with positron emission tomography (PET) (theranostic), we wanted in this work to assess the types of low energy electron interactions with DNA (dissociative electron attachment (DEA), excitation and ionization) and their relative importance in causing DNA breaks as a function of electron energy. In a second aim, we wanted to reproduce with Monte Carlo simulation the yield of SSB and DSB produced by low energy electrons from an experimental setup of DNA in a vacuum [22], [23]. Although GEANT4-DNA enables us to simulate water radiolysis and the effects of its species in DNA damage, which makes the simulation more realistic, this part of the simulation was neglected in the present work to assess the direct effect of the electrons on DNA and to compare our results with the previously published results [22], [23]. For this purpose, we simulated a model of an atomistic DNA, the B-DNA configuration, where each monomer was a nucleotide composed of a nucleic base (adenine, cytosine, guanine, and thymine) coupled to a deoxyribose molecule, which in turn is linked to a phosphate group, thus forming 1122 bases of length 30 nm [8], [15]. We used several electron energies and compared our results with the experimental results from [22], [23]. The main rationale of this study was the assessment of the relative importance of the different interaction mechanisms of low energy electrons in B-DNA configuration.

## 2 Materials and Methods

The geometry of DNA was created using the PDB files from the Protein Data Bank (PDB), which provides various data for many molecular models (https://www.rcsb.org/) [7], [9]. These data describe the macromolecule atomic localization, i.e., the Cartesian coordinates of the atoms in a three-dimensional coordinate system. Furthermore, the PDB dataset provides a simple notation for building the DNA molecules in space, as well as many additional atomic details.

The DNA geometry chosen for this work was the B-DNA, which was formed of two anti-parallel strands wrapped around one other to form a double helix. Each strand is a polymer known as a polynucleotide. Each monomer is a nucleotide, which is composed of a nucleic base (adenine, cytosine, guanine, or thymine) coupled to a deoxyribose molecule, which in turn is linked to a phosphate group.

During the geometry simulation, a partially condensed DNA structure was chosen to represent the cells in vivo. For such needs, the atomic positions have been extracted one by one from a PDB file, giving the atomic description of a DNA molecule containing 1122 base pairs with a final length of 30 nm, thus making 2 × 1122 DNA strands. Each atom was represented by a sphere whose radius is equal to the radius of van der Waals [24], and the molecules were constructed using the G4UnionSolid class integrated into GEANT4 [25]. This routine allows us to properly combine two solids of different shapes to create a new shape. Using this method, we have placed the molecules one by one by placing the spheres representing their atoms. Each molecule was represented by a set of spheres that have been well united. The phosphate group, for example, was represented by a union of 5 spheres, 1 atom of phosphorus and 4 atoms of oxygen. The spheres were filled with materials that have the same density as the DNA molecules to which they belong. This technology not only makes geometry simulation easier, but also makes it faster to precisely identify the interactions of atoms and molecules with the incident and secondary particles.

We simultaneously simulated six DNA targets with a length of 30 nm, all were placed in a vacuum cube with an edge of 400 nm, and an isotropic source was set at 40 nm from the six DNA targets. The primary particles were mono-energetic electrons produced according to a solid angle to cover all the six molecules of the DNA. The energies used were 4 eV, 6 eV, 10 eV, 15 eV, 20 eV, 30 eV, 50 eV, 100 eV, 200 eV and 500 eV. In each simulation, the DNA targets were irradiated with $10^{7}$ electrons of the same energy, and each simulation was repeated 10 times to calculate the mean value and the standard deviation for uncertainty calculation. The energy deposit was counted for each interaction type and at each position x, y, z in the volume of DNA, thus localizing the atoms where the interactions occurred. Based on the number and types of interactions at the atomic level, and by comparison with experimental measurements [26], [27], [28], [29], the number of DNA single strand and double strand breaks were calculated.

The Monte Carlo code used in this work was GEANT4 which is provided in the form of a toolkit containing the elements necessary to simulate the particle transport and interactions (https://indico.cern.ch/event/647154/contributions/2714212/attachments/1529029/2397032/BookForApplicationDevelopers.pdf).

In the present study, the use of new material in GEANT4-DNA necessitated the incorporation of new cross-sections prior to the computation of the mean free path and interactions of the particles. We used the physical processes in GEANT4-DNA to simulate all possible interactions between electrons and DNA, i.e., ionization, elastic interaction (no energy transfer), vibrational and electronic excitations (grouped under the name of excitation), and dissociative electron attachment (DEA).

For the two types of ionization and elastic interactions, we used the two models G4DNAPTBElasticModel and G4DNAPTBIonizationModel which were introduced in GEANT4-DNA by Bug et al. [30]. A simplified model of the Auger effect has also been presented. Any ionization of carbon or oxygen atom results in the release of an Auger electron, whereas ionization of a nitrogen atom results in the emission of two electrons.

The interaction by excitation was also defined by introducing a new cross-section for the 6 DNA molecules: the four bases plus deoxyribose and phosphate group. When an excitation interaction occurs, among the permitted levels of excitations, we randomly selected one from them to calculate the deposited energy.

As previously stated, electrons with energies lower than the vibrational excitation energy can induce the DNA strand breaks through DEA. For that purpose, a new cross-section has been introduced to properly identify the effects of these electrons with energies below 10 eV.

**Figure 1:**
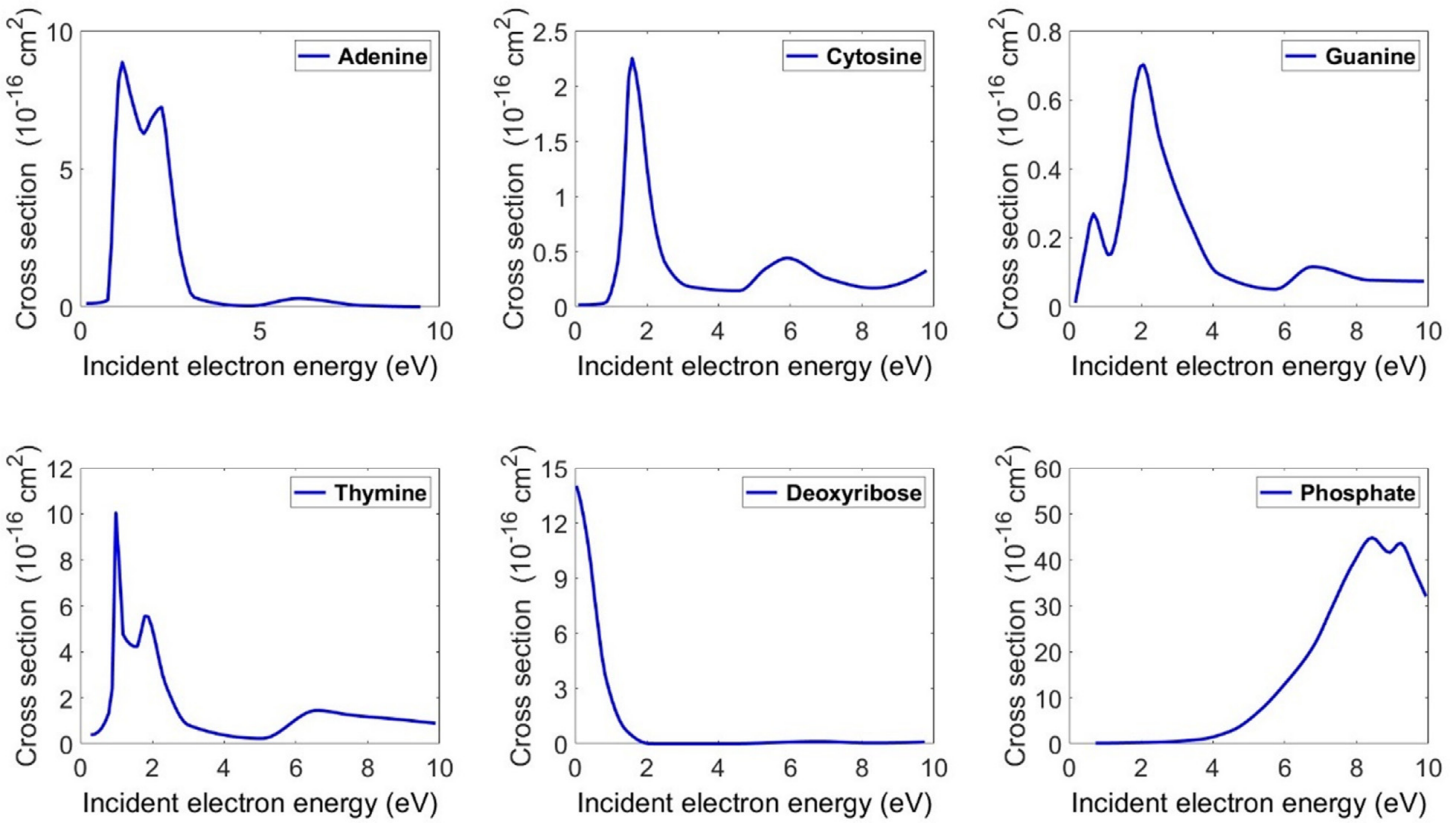
Deduced DEA cross-sections from experimental studies [26], [27], [28], [29]. These cross-sections were used in our simulations.

The previous experimental studies from which we extracted the cross-sections were those of Abdoul-Carime et al. (2005), Denifl et al. (2004), Ptasińska et al. (2004), and Pan and Sanche (2006) [26], [27], [28], [29]. The first two works gave a detailed study of the DEA for adenine, cytosine, guanine and thymine isolated in the gas phase. Using these works, we could directly extract the cross-sections of the four bases. On the other hand, we extracted the DEA cross-sections of the phosphate group and the deoxyribose sugar fraction from measurements performed by Ptasińska et al. (2004) and Pan and Sanche (2006) [28], [29]. The deduced cross-sections of these molecules are shown in Fig. 1. Our calculated cross-sections were very similar to those reported in [5]. The cross-sections of the other electron interactions are described in [30], [31]. According to these cross-sections (Fig. 1), the electrons can interact with the DNA bases in the energy range below 4 eV. Meanwhile, the DEA is favored in the phosphate group in DNA strand molecules, as seen in Fig. 1, which shows a high peak in the electron energy range between 4 and 10 eV. However, the deoxyribose can be dissociated by electrons with lower energy between 0 and 2 eV.

In this work, we considered the DEA as a competitive interaction with the electronic excitation according to the cross-sections discussed above. When we had a dissociative electron attachment, we assumed a break of the molecule. As a result, the electron is no longer tracked.

In the present study, in addition to the determination of the cross-sections from experimental reports and their incorporation in GEANT4, we used a new approach to count SSB and DSB at the DNA level. We assumed that the breakage threshold strongly depends on the type of interaction. For this purpose, we considered the three main types of interactions (dissociative electron attachment, excitation and ionization), each interaction type has a way of inducing a DNA breakage according to the cross-sections described above. First, given that the ionization is a key factor in DNA damage, we assumed that an SSB is formed at the DNA level by ionization if the interaction occurs accompanied by a local energy deposition beyond the ionization threshold of 10 eV [20]. Alternatively, we considered that the vibrational excitation has an important role in the dose deposition, but it does not represent a dissociative pathway of DNA. Following that, we assumed that the DEA interaction is a direct DNA dissociation pathway, and that if we have a DEA interaction, it directly leads to a single break in the DNA. We mention also that the cross-sections of the vibrational excitation and the electronic excitation were commonly used from the same table. An energy deposit by vibrational excitation was ignored and this occurs at low energy, while an energy deposit by electronic excitation was translated to an SSB if this energy transfer is greater than the excitation energy threshold of the target molecule.

Strand breaks are most typically seen in the DNA backbone (phosphate and 2-deoxyribose) and are caused by the phosphate-deoxyribose bond or, less commonly, the base-deoxyribose bond. The breaks at the DNA base level can also cause strand break under the effect of low energy electrons by a charge transfer from the DNA bases to the strand molecules, however in this work, we only considered the direct effects on DNA strands. For that purpose, we assumed that an SSB is defined as a break under the energy deposition as discussed above, and a DSB is defined as two SSBs located on two opposite strands with a distance of less than 10 nucleotide pairs [7], [32]. The authors in [7], [32] justified the occurrence of an SSB as an equivalent energy transfer to the first excitation energy level of 8.22 eV in liquid water.

The first parameter calculated was the dose deposited by all interactions (in Gy) for each given energy beam. The second parameter calculated is the DNA strand break yield per incident electron, by dividing the total number of breaks by the total number of incident electrons. The yield of single strand breaks (YSSB) and double strand breaks (YDSB) were also calculated in the units of strand breaks per Gray per Dalton to make a relationship between the geometry of the DNA, the deposited dose, and the DNA weight [21], [33].

$$Y_{\mathrm{SSB}(DSB)}=\frac{\mathrm{Number}\;of\;SSB\;\left(DSB\right)}{Dose\left(Gy\right)\times DNA\;weight\;\left(Dalton\right)}$$

Finally, the number of strand breaks was determined under the effect of the incident electron energy, and at the end we compared the three electron interactions, to determine the dominant breaking mechanism for every given energy.

**Figure 2:**
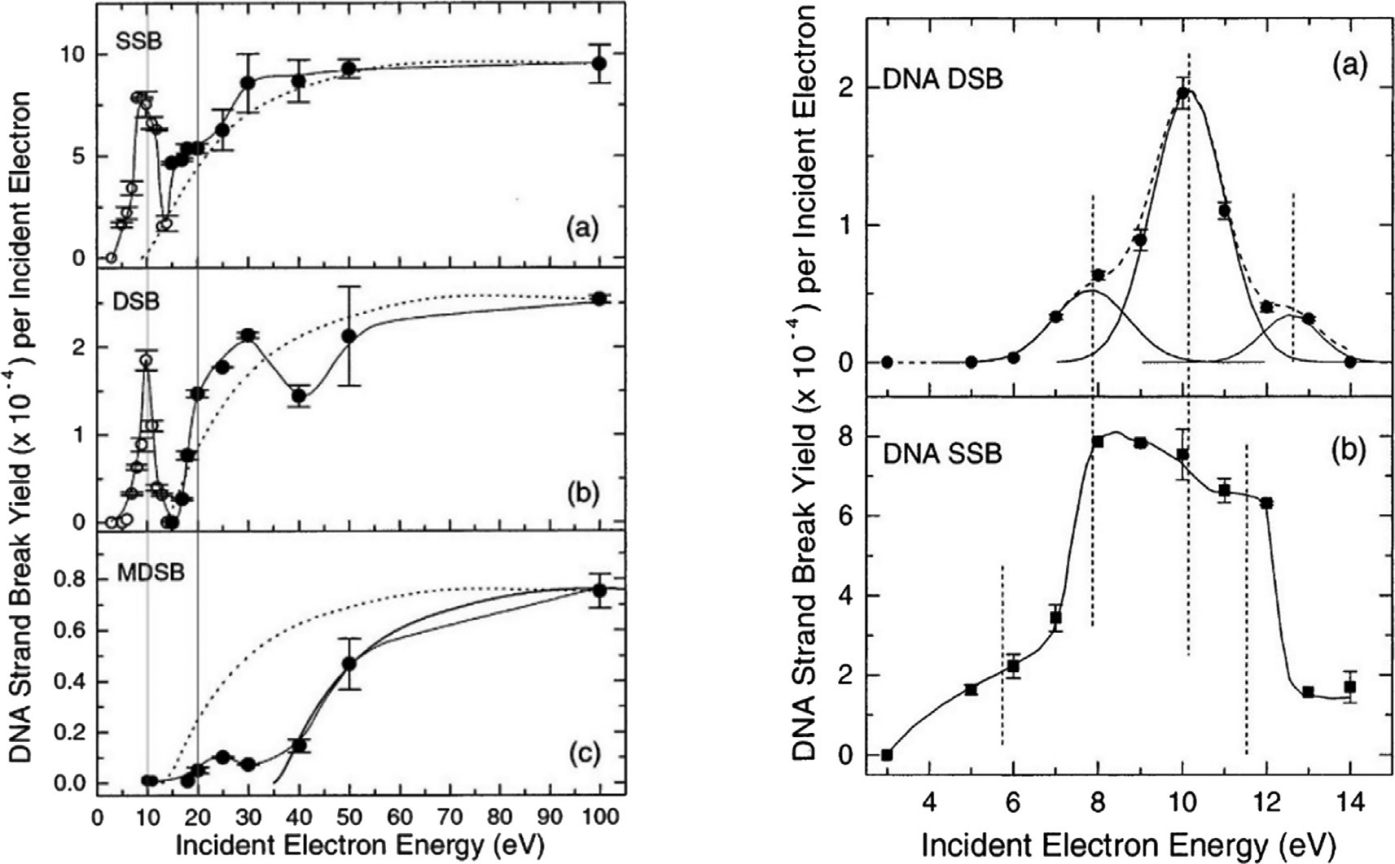
Left: DNA SSB and DSB as a function of electron energy. MDSB stands for multiple DSBs and this was not simulated in our work. The light vertical lines are guides indicating the 10 eV and 20 eV electron energy. Right: SSB and DSB fitted with gaussians to isolate electron interaction effects on DNA. Reprinted with permission from [23]. Copyright 2022 from the Journal of the American Chemical Society.

Because we compared our results with those in the experiments in [22], [23], we briefly describe this experimental setup. The study was conducted with an electron accelerator. The authors used a DNA with 3197 base pairs. The DNA was set on a tantalum support inside an upper high vacuum chamber and irradiated with an electron beam of energy varying between 5 eV and 1 keV. A detailed description of the experimental setup can be found in [34]. Fig. 2 depicts the results from such experiments showing the yields of SSB and DSB, and the fitting of these results in an attempt to identify the partial contribution from the different electron interactions.

## 3 Results

**Figure 3:**
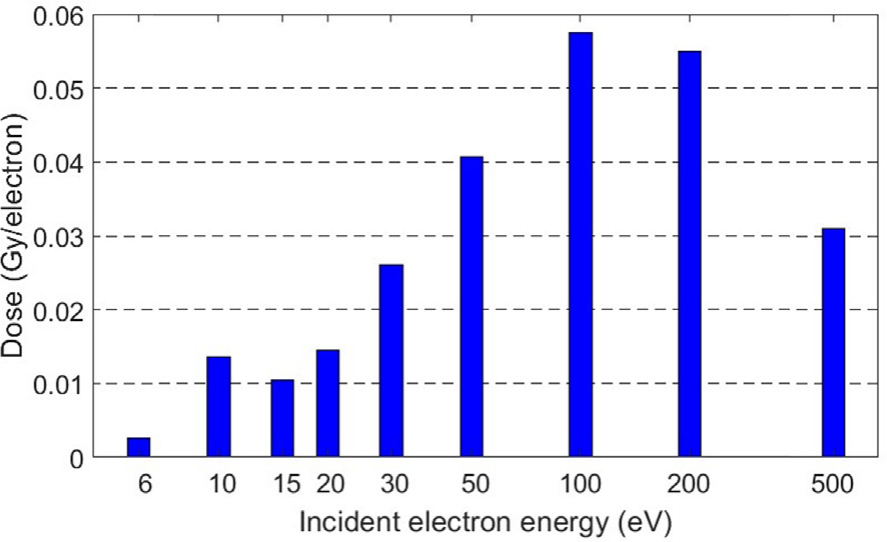
Deposited dose in DNA in Gy per incident electron as a function of the simulated electron energies. For the energies simulated, the electrons with 100 eV produced the highest dose deposited in DNA. A small peak appears at 10 eV corresponding to the dissociative electron attachment cross-section. The x-axis was plotted in a log scale to visually separate the graph bars. Please note that, not all the simulated electrons interact with DNA, and not all the interacted electrons in DNA deposit their total energy.

Fig. 3 shows the deposited dose in DNA increasing with the electron energy until a maximum dose of $5.710^{5}$ Gy at 100 eV (normalized here by dividing by $10^{7}$, the number of simulated electrons), then progressively decreasing at higher electron energies. Each data bar in this graph is the result of the simulation at the corresponding energy. We notice a small peak at 10 eV, which corresponds to the high dissociative electron attachment cross-section. The deposited dose for each beam depicts the amount of energy deposited through the three interaction types for every simulated energy. The deposited dose contains all doses deposited by the primary and secondary electrons.

**Figure 4:**
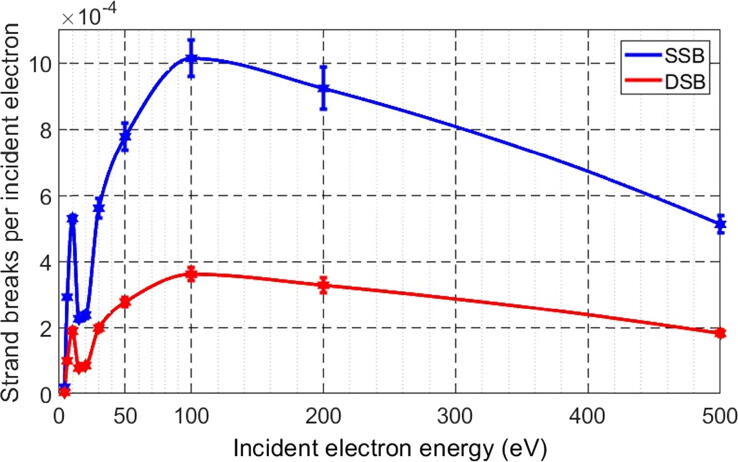
DNA strand breaks for SSB and DSB per incident electron as a function of electron energy.

Fig. 4 shows the yields of SSB and DSB per incident electron under the effect of each energy beam between 4 eV and 500 eV. The error bars correspond to the standard deviation for each given energy deduced from simulations repeated 10 times in the same configuration. From these figures, we can clearly distinguish the properties of each energy. In general, the two curves have the same features and shape, with the quantity of SSB being nearly 2.5 times that of DSB. We recall that in these simulations, a DSB was counted if the two SSB were produced in opposite DNA strands within a distance of 10 DNA base pairs. It has been reported that DSB production from two different primary particles is unlikely (<1%) [35].

The creation of SSB and DSB starts at the lowest simulated energy of 4 eV. The amount of SSB and DSB rapidly increases to reach a local maximum of $5.310^{-4}$ SSB per electron and $1.910^{-4}$ DSB per electron at 10 eV because of the cross-section of DEA, which dominates in this energy range. Subsequently, the quantities of breaks decrease to make a minimum between 13 eV and 18 eV due to the disappearance of the DEA. They thereafter increase to arrive at the highest maximum of $10.1510^{-4}$ SSB per electron and $3.6210^{-4}$ DSB per electron for the electrons of 100 eV.

**Figure 5:**
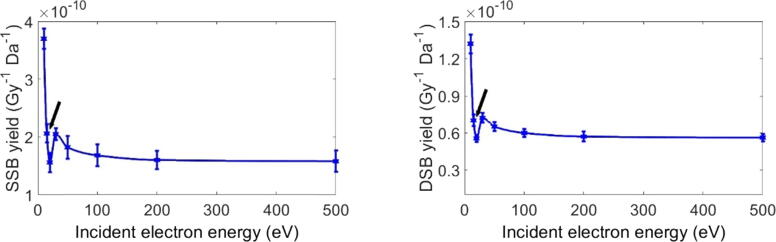
Yields of SSB and DSB per Gy and per Dalton for the indicated electron energies. The arrow indicates the 15 eV-data point. A few electron interactions depositing low doses at very low electron energy increased the yield (dividing by low dose values).

Fig. 5 shows the yields calculated for single-strand breaks ($Y_{\mathrm{SSB}}$) and double-strand breaks ($Y_{\mathrm{DSB}}$) in units of breaks per Gy and per Dalton for the simulated energy from 10 eV to 500 eV. Due to the small amount of the deposited dose by the electrons with the lowest energy, a high yield appears for energies less than 15 eV (black arrow in Fig. 5). This energy corresponds to DNA sensitivity to low energy electrons, which implies that even if the deposited dose is too small, the low energy electrons can cause SSB and DSB. Thereafter, at 20 eV, the SSB and DSB yields increase to a maximum near 30 eV, after that the yields start to gradually decrease to make a plateau after 100 eV.

The percentage of the number of single strand breaks under the effect of each electron energy beam (number of single breaks divided by total number of strands, i.e. by 2 × 1122) was found to have two phases (figure not shown). In the first phase between 4 eV and 30 eV, the number of strand breaks started from around 8% at 4 eV, noting that 4 eV was the lowest electron energy simulated, then when the energy of the electrons increased, the breaking yield also increased and showed a peak at 10 eV, then it decreased to reach a minimum of almost 63.8% of strand breaks for the electrons having an energy of 15 eV. The second phase, depicting the number of strand breaks, increased with energy to reach values above 80% at energies above 100 eV. This means that electrons with energy greater than 100 eV were the most damaging to DNA, which is expected since when using such energies, these electrons create secondary electrons that have low energies below 50 eV. However, as a counterbalancing effect, the probability of these incident electrons interacting in DNA is less when the energy increases.

**Figure 6:**
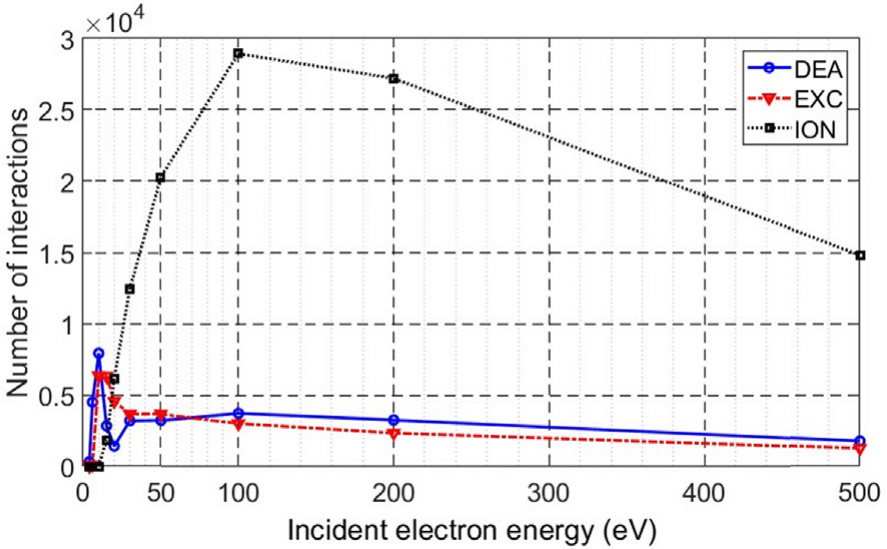
The number of each type of interaction for each simulated electron energy beam. DEA is for dissociative electron attachment, EXC is for excitation and ION for ionization.

Fig. 6 shows the quantity of each type of interaction and for each electron energy simulated. For the simulation with 4 eV, we notice that the 10 million electrons made 316 DEA events, whereas the 6 eV electron beam generated 4484 DEA events and a small quantity of excitation events. This reflects the fact that DEA is the dominant interaction in the energy below 10 eV. The values for the incident electron energies 4, 6, and 10 eV, the DEA, excitation and ionization were respectively 316, 4484, and 7916; 0, 100, and 6332; 0, 0, and 0 interactions.

The electrons of 10 eV performed both types of interactions, DEA and excitation. However, above 15 eV, we noticed that ionization began to occur. For the 15 eV electron beam, the excitation presented the major interaction, 6307 interactions versus 2830 for DEA and 1811 for ionization, while for the 20 eV, ionization showed the major interaction, indicating that the ionization cross-section was greater than those of the other interactions: 6143 interactions versus 1410 for DEA and 4548 for excitation. We observe in Fig. 6 that for beams with energies greater than 20 eV, the dominant interaction remains the ionization, which reaches a maximum for beams with energies of 100 eV. Furthermore, when the energy exceeds 30 eV, the primary electrons can only perform the ionization events and the other types of interaction came from the secondary electrons which were released as a result of DNA ionization.

Apart from the comparison with the experimental results in Fig. 2 where our results display a great similarity, we have also compared our results with the simulations of Nikjoo et al. and Charlton et al. in Table 1 [21], [36]. Although the configuration of these simulations and the energy threshold on secondary electron tracking was not exactly similar to those in our work, the simulations mainly targeted SSB and DSB as in our work, and their results are comparable to our simulation. We compared the yields calculated for single strand breaks ($Y_{\mathrm{SSB}}$) and double strand breaks ($Y_{\mathrm{DSB}}$) per Gy per Dalton for electron beams of 100 eV and 300 eV (280 eV in [36]), and the results are presented in Table 1. It can be seen that the calculated values for $Y_{\mathrm{SSB}}$ are not very dissimilar, with our value at 300 eV located between those of the other two works. However, the values of $Y_{\mathrm{DSB}}$ appear biased in comparison to the other works, which might depend on the definition of the occurrence of the DSBs in liquid water, and the determination of the cross-sections.

**Table 1:** Comparison of yields of SSB and DSB between the present work and works from Nikjoo et al. and Charlton et al. [21], [36].

E (eV)	Y_SSB_ ($Gy^{-1}Da^{-1}\times 10^{-10}$)			Y_DSB_ ($Gy^{-1}Da^{-1}\times 10^{-11}$)			Total SSB/Total DSB	
	Our work	Nikjoo et al.	Charlton et al.	Our work	Nikjoo et al.	Charlton et al.	Our work	Nikjoo et al.
100	1.55	2.5	–	5.8	1.5	–	3.4	–
300	1.6	2.5	1.36	5.7	2.3	2.64	2.8	3.4

## 4 Discussion

In radiation therapy by external radiation beam or by radionuclide administration, the ultimate process of radiation interaction with DNA is mostly achieved by low energy electrons. Radionuclides targeting DNA of tumor cells can be associated with monoclonal antibodies to specifically penetrate tumor cell nuclei [37].

In order to use internal radiation therapy, imaging of the patient is first conducted to locate the tumor, its density, its volume and its surrounding normal tissues. Although the tumor is macroscopic and the interaction of DNA is sub-microscopic, the dose-equivalent to SSB and DSB can be extracted from GEANT4-DNA and normalized in such a way to discriminate the dose to DNA from other doses in the tissues [38]. To date, it is very demanding in space and time to develop the geometry even at the level of a single tumor cell in order to locate its DNA. The experimentation and the simulation of isolated DNA until now have been conducted in several environment configurations, such as in water or in the presence of gases.

The knowledge of the types of interactions with DNA is of great importance in order to select the appropriate isotopes or external beams. Validating a simulation by comparison with an experimental measurement allows, in turn, to estimate the impacts of the individual types of interactions as a function of energy, which cannot be done by experimentation. Therefore, in the present work, we compared our results to the studies conducted experimentally in [23] in addition to the extraction of the cross-sections from the experiments on DNA [26], [27], [28], [29], and we provided the types of interactions individually as a function of energy induced by primary beams (Fig. 6).

The SSB and DSB as reported in Fig. 2 integrate the global effects of all electron interactions and all electrons with various energies set in motion by the incident electrons, including the recoiled incident electrons on the Tantalum in the experimental setup [23]. Thus, it is not possible to isolate the SSB and DSB yields induced by the primary electrons only, or by a given energy of primary or secondary electrons. In the present simulations, by considering an isolated DNA in a vacuum in a similar fashion as in [23], the yields of SSB and DSB were found to be comparable with the peak at 10 eV in Fig. 2, corresponding to the same electron energy of 10 eV in Fig. 4, followed in both cases by a depression at 15 eV. Despite the DSB yield in Fig. 2 being generated by different electron interactions, while in our simulation, a DSB was counted as two opposite strands breaks within a distance of 10 DNA base pairs, the yields of SSB and DSB appear very comparable, as can be seen by the peak at 10 eV for SSB, which is around $8\times 10^{-4}$ in Fig. 2 and of $5.3\times 10^{-4}$ in Fig. 4, and for DSB, the values are, respectively, around $1.8\times 10^{-4}$ and $1.889\times 10^{-4}$. The slight difference between the SSB values might be due to the recoiled electrons on the Tantalum, while these electrons are less prominent to provoke DSBs. In addition to these similarities with the experimental measurement, Fig. 6 depicts the number of interactions for each simulated type of interaction, DEA, excitation and ionization, as a function of energy, allowing us to isolate each of the three phenomena. Noting that the highest energies also contribute to the DEA and excitation interactions by means of their secondary electrons.

The comparison of the present simulation with the simulation made by Nikjoo et al. [21], from which the values are reported in Table 1, should take into account the difference in the simulation environment. Nikjoo et al., in addition to the direct effect of the electrons, they counted the OH radical interactions. They used electron energies from 100 eV to 4.5 keV, which is higher than the energies used in our simulation, and they tracked the electrons until 10 eV in liquid water. They also limited the production of SSB to an energy threshold of 17.5 eV for electrons of 300 eV.

The values from the work of Charlton et al. reported in Table 1 were deduced from a model depending on the energy deposited, and these values were compared to the experimental values [36]. The DNA was considered as a cylinder of diameter 2.3 nm, the DSB provoked by two different electrons were not counted, an energy threshold of 17.5 eV was used, and only one SSB and one DSB were counted in a DNA base pair to conform to the experimental results. The values from Charlton et al.'s reference inserted in Table 1 were read from Fig. 6 of this reference.

In the present work, one of the important advantages is that the action of the DEA causes DNA DSB which is expected to be profitable for local cancer treatment with low energy electrons from β or Auger electron emitters, avoiding the use of high energy particles, which are harmful to normal cells.

On the other hand, the present simulations considered an isolated atomistic DNA molecule in order to develop the model and to compare it with the previously published experimental and simulated results. Even if the present results are in accordance with the experimental measurements in [23], the decision to translate energy transfer to SSBs remains open to discussion. For each interaction considered in this work, we used thresholds to consider the occurrence of an SSB: DEA was directly translated into an SSB; excitation depended on the energy threshold of molecular excitation; ionization gave an SSB above 10 eV energy transfer; and finally, a DSB is the sum of two SSBs within a distance equivalent to 10 base pairs. These thresholds were used according to other publications as described above in the section of Materials and Methods. The assumption of the molecules made by bonds of spheres with van der Waals radii also introduces parts of uncertainties. Even the side of DNA exposed to the radiation and inclusion of base breaks can affect the overall DNA damage.

Moreover, the inclusion of water species produced by the incident radiation should produce more realistic results. The inclusion of a whole cell, or more consistently, the inclusion of a tissue like a volume of a tumor makes the calculations too complicated to design with many parameters, and too lengthy for the simulation. One of the many possibilities is to perform simulations like the one presented here, which can be validated with experiments, and by grouping them in Deep Learning processes such partial simulations targeting specific phenomena, Deep Learning might then generate accurate results faster than the complex Monte Carlo simulations.

## 5 Conclusion

We reported in this paper the rates of the interaction types in DNA as a function of electron energies: dissociative electron attachment, excitation and ionization. The single strand and double strand breaks of DNA were found to be comparable to those obtained with experimental setups. The dissociative electron attachment was found to be the only present at energies below 10 eV of electron energy. Excitation appears around 10 eV, while the ionization becomes dominant at 20 eV and above.

## Declaration of Competing Interest

The authors declare that they have no known competing financial interests or personal relationships that could have appeared to influence the work reported in this paper.
